# Caffeoylquinic Acids Are Major Constituents with Potent Anti-Influenza Effects in Brazilian Green Propolis Water Extract

**DOI:** 10.1155/2011/254914

**Published:** 2011-03-01

**Authors:** Tomohiko Urushisaki, Tomoaki Takemura, Shigemi Tazawa, Mayuko Fukuoka, Junji Hosokawa-Muto, Yoko Araki, Kazuo Kuwata

**Affiliations:** ^1^Nagaragawa Research Center, API Co., Ltd., 692-3 Nagara, Yamasaki, Gifu 502-0071, Japan; ^2^United Graduate School of Drug Discovery and Medical Information Sciences, Gifu University, 1-1 Yanagido, Gifu 501-1193, Japan; ^3^CREST, Japan Science and Technology Agency, 4-1-8 Honcho, Kawaguchi, Saitama 332-0012, Japan; ^4^Center for Emerging Infectious Diseases, Gifu University, 1-1 Yanagido, Gifu 501-1194, Japan

## Abstract

Influenza A viral infections reached pandemic levels in 1918, 1957, 1968, and, most recently, in 2009 with the emergence of the swine-origin H1N1 influenza virus. The development of novel therapeutics or prophylactics for influenza virus infection is urgently needed. We examined the evaluation of the anti-influenza virus (A/WSN/33 (H1N1)) activity of Brazilian green propolis water extract (PWE) and its constituents by cell viability and real-time PCR assays. Our findings showed strong evidence that PWE has an anti-influenza effect and demonstrate that caffeoylquinic acids are the active anti-influenza components of PWE. Furthermore, we have found that the amount of viral RNA per cell remained unchanged even in the presence of PWE, suggesting that PWE has no direct impact on the influenza virus but may have a cytoprotective activity by affecting internal cellular process. These findings indicate that caffeoylquinic acids are the active anti-influenza components of PWE. Above findings might facilitate the prophylactic application of natural products and the realization of novel anti-influenza drugs based on caffeoylquinic acids, as well as further the understanding of cytoprotective intracellular mechanisms in influenza virus-infected cells.

## 1. Introduction

Worldwide swine-origin H1N1 influenza virus infection became a pandemic in 2009 [1]. Two of the viral proteins, neuraminidase (NA) and the M2 ion-channel protein, are the primary targets of current influenza antiviral drugs [2]. Unfortunately, there is already widespread resistance to both drug classes [3]. Recently, a novel drug candidate targeting RNA polymerase has been reported [4]. Despite the recent advances in influenza therapies, direct viral drug targets are generally limited; therefore, we must consider new strategies in drug development for the mitigation of influenza virus infection.

Propolis is a resinous substance that is collected by honeybees from plant sources and is thought to play a protective role against potential predators. Propolis has been used in folk medicine and has been reported to possess therapeutic and prophylactic effects against inflammation, heart disease, diabetes mellitus, hepatotoxicity, and cancer [5, 6]. Antiviral activity of propolis were demonstrated comprising anti-BBMV [7], anti-HSV [8–12], anti-poliovirus [13], anti-r IBDV [14], anti-reovirus [14], anti-HIV [15–17], and so on [14, 18–20]. There are numerous reports regarding the anti-influenza virus activity of propolis [21–24]. However, no effective constituents have been isolated from propolis for influenza virus treatment or prophylaxis.

The constituents of propolis are greatly influenced by its production area and plant origin. Currently, propolis is classified into many different type such as European (poplar type), Brazilian (*Baccharis* type, derived from Alecrim; *Baccharis dracunculifolia* (Compositae) [25]), Cuban, and Taiwanese type [26]. Specially, both poplar type and *Baccharis* type of propolis have been deeply studied. In Japan, Brazilian green propolis, which is *Baccharis* type and originates from Minas Gerais, is most popular. In other words, health supplement utilizing Brazilian green propolis occupies mostly in Japanese propolis health food market today. Moreover, recent reports revealed that propolis collected from very specific and limited areas in southern Brazil has an anti-influenza effect [24]. Thus we chose the Brazilian green propolis from Minas Gerais for study.

In this paper, we have identified the major constituents with the anti-influenza virus activity in Brazilian green propolis and have further investigated the mechanisms of these activities using a combination of cell viability and real time PCR assays.

## 2. Methods

### 2.1. Cells, Viruses, and Compounds

Madin-Darby canine kidney cells (MDCK cells) was provided by Professor Hideto Fukushi, United Graduate School of Veterinary Sciences, Gifu University. The influenza A virus strain A/WSN/33 (H1N1) was provided by Prof. Yoshihiro Kawaoka, the Institute of Medical Science, the University of Tokyo. Minimal essential medium (MEM, Wako Pure Chemicals, Osaka, Japan; or Invitrogen, Carlsbad, California), fetal bovine serum (Equitech-bio, Inc., Kerrville, TX, USA), phosphate buffered saline (PBS) (Gibco, Rockville, MD), penicillin and streptomycin (Gibco, Rockville, MD), and Cell Counting Kit-8 (Dojindo, Kumamoto, Japan) were used. Water extract of Brazilian green propolis, which was collected in the state of Minas Gerais (PWE, product name: Proapi), was supplied by API Co., Ltd. Chlorogenic acid and caffeic acid were purchased from Tokyo Chemical Industry Co., Ltd. (Tokyo, Japan), and quinic acid was purchased from Nacalai Tesque, Inc. (Kyoto, Japan). 3,4-Dicaffeoylquinic acid (3,4-diCQA), 3,5-dicaffeoylquinic acid (3,5-diCQA), 4,5-dicaffeoylquinic acid (4,5-diCQA), and 3,4,5-tricaffeoylquinic acid (3,4,5-triCQA) were isolated from the propolis, as previously described [27]. The 3,4-dicaffeoylquinic acid (3,4-diCQA), 3,5-dicaffeoylquinic acid (3,5-diCQA), 4,5-dicaffeoylquinic acid (4,5-diCQA), and 3,4,5-tricaffeoylquinic acid (3,4,5-triCQA) were 85.5%, 90.0%, 51.4%, and 87.4% pure, respectively. The purity of 4,5-diCQA has decreased rapidly by degradation during storage. Its purity was 90% or more just after the purification.

### 2.2. In Vitro Anti-Influenza Virus Assay and Cytotoxicity Assay

To assess anti-influenza activity, MDCK cells (2 × 10^5^/well) were cultured in MEM (Wako) that contained 10% fetal bovine serum, 60 U/mL of penicillin, and 60 *μ*g/mL of streptomycin for 24 hrs on 96-well plates, washed with PBS, and infected with 20 to 200 TCID_50_ A/WSN/33 virus in the presence or absence of compounds. Compounds were added almost simultaneously in an assay medium, MEM (Invitrogen) that was supplemented with 1% BSA, 1% DMSO, and 6.25 *μ*g/mL trypsin. Cell culture were maintained without medium exchange at 37°C, 5% CO_2_ for 48 hrs. Culture supernatants were collected 48 hrs after infection for real time PCR assay. Remaining cells were then washed twice with PBS, and the antiviral effects of the compounds were evaluated using a cell viability assay (WST-8 (2-(2-methoxy-4-nitrophenyl)-3- (4-nitrophenyl)- 5-(2,4-disulfophenyl) -2H tetrazolium monosodium salt), Cell Counting Kit-8) to measure the probability of survival [28]. The EC_50_ (half maximal effective concentration) value on the cell survival is determined by curve fitting method using GraphPad Prism for Windows (Version 5.02, GraphPad Software, Inc.) under a nonlinear regression curve fitting in which the maximum response value was set to 100% or less. Cytotoxicity was assessed using assays with no viral infection.

### 2.3. RNA Extraction and Quantitative Real-Time PCR Assay

Viral genomic RNA was extracted from the supernatants of the culture using the High Pure Viral RNA Kit (Roche Diagnostics, Mannheim, Germany) according to the manufacture's protocol. A 200-*μ*L aliquot of supernatant was dissolved in a lysis buffer that contained poly-A that was bound to a glass fiber column. RNA was eluted from the column using 50 *μ*L of nuclease-free water. cDNA was synthesized from 2 *μ*L of the eluate by using a PrimeScript RT Reagent Kit (Perfect Real Time, Takara Bio Inc., Otsu, Japan) and random hexamer as reverse transcription (RT) primers according to the manufacture's protocol. Quantitative real-time PCR for H1N1 was performed. The RT reaction product was amplified by using SYBR Premix Ex Taq (Takara Bio Inc., Otsu, Japan) and Thermal Cycler Dice Real Time (Takara Bio Inc., Otsu, Japan) according to the manufacturer's protocol. H1N1-specific primers were selected using Primer Express Software (PE Applied Biosystems) and based on the polymerase basic protein 1 gene (PB1). The sequences of the primer sets included 5′-GATGGACAACAAACACCGAAACT-3′ as the forward primer and 5′-TACACAATGTTTGGGCATAACC-3′ as the reverse primer. Quantitative RNA levels relative to the control, which were derived from the supernatant of the culture by adding vehicle and H1N1-expressing infected cells, were estimated using the standard curve of serial dilution.

### 2.4. Statistical Analysis

Data were analyzed by one-way analysis of variance (ANOVA), followed by a Bonferroni multiple comparison test using JSTAT, version 12.6, for Windows (Masato Sato, Japan).

## 3. Results

### 3.1. Major Components in PWE

**Figure 1:**
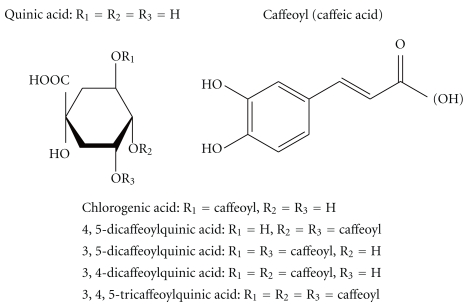
Chemical structure of the caffeoylquinic acids that were derived from the propolis used in this study.

Figure 1 shows the chemical structure of several caffeoylquinic acids that are the primary components of the propolis water extract (PWE) and include chlorogenic acid, 3,4-diCQA, 3,5-diCQA, 4,5-diCQA, and 3,4,5-triCQA. Caffeoylquinic acids are phenolic acids and esters of polyphenolic caffeic acid (its number is from one to three) and quinic acid (both also shown in Figure 1(a)). The percentage concentration of each component (Table 1) has been already reported [27, 29]. Concentrations of chlorogenic acid, 3,4-diCQA, and 3,5-diCQA acid are relatively high, that is, 2.7%–3.6%, 3.3%–6.1%, and 4.3%–4.9%, respectively. Other components of PWE such as *p*-coumaric acid [27, 29] were not shown here.

### 3.2. Antiviral Effects of Each Ingredient in The PWE

**Figure 2:**
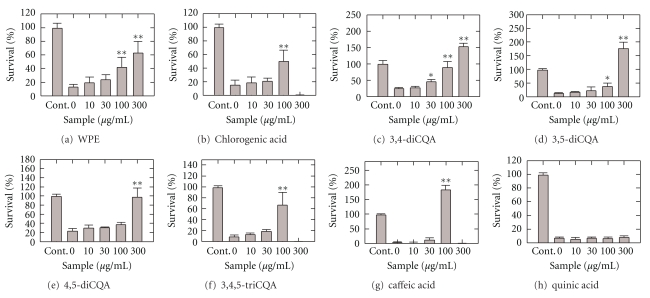
The antiviral effect of PWE and its components in MDCK cells infected with influenza A virus. Cell viability is plotted as a function of the concentration of applied sample (*μ*g/mL). Results are presented as mean value ± standard deviation; *n* = 6. *P < .05 and **P < .01 in comparison to 0 *μ*g/mL.

The overall impact of the PWE on the cell survival rate in WSN/33 infected MDCK cells is depicted in Figure 2(a). Cell viability of MDCK cells was inhibited under the presence of virus. In the presence of 100 to 300 *μ*g/mL of the PWE, the percentage of the cell viability significantly (*P* < .01) increased. The EC_50_ value on the cell survival by PWE was 183.1 ± 6.0 *μ*g/mL (mean ± standard error). We then investigated the individual ingredients in the PWE so as to identify the specific ingredient that was responsible for this effect. As can be observed in Figures 2(b)–2(h), chlorogenic acid, 3,4-diCQA, 3,5-diCQA, 4,5-diCQA, 3,4,5-triCQA, and caffeic acid were effective. Quinic acid was ineffective. The EC_50_ values of the cell survival by each component shows that 3,4-diCQA was the most potent (EC_50_ = 81.1 *μ*M (41.9 *μ*g/mL)) in the tested compounds (Table 2). We also tested similar activity of the other components of PWE, such as *p*-coumaric acid, artepillin C, baccharin, drupanin, and kemferide (details not shown). These compounds did not have any cell surviving activity of viral infected cells (data not shown). Thus, it is highly probable that 3,4-diCQA is the predominant chemical that is responsible for the anti-influenza effect of the PWE.

### 3.3. Cell Toxicity of Each Ingredient in The PWE

**Figure 3:**
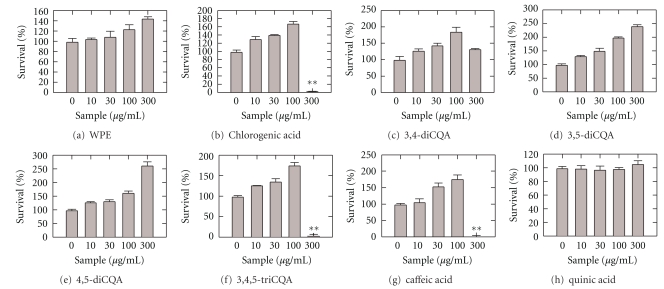
The cytotoxicities of PWE and its components in MDCK cells. Cell viability is plotted as a function of the concentration of applied sample (*μ*g/mL). Results are presented as mean value ± standard deviation; *n* = 6. *P < .05 and **P < .01 in comparison to 0 *μ*g/mL.

Chlorogenic acid, 3,4,5-triCQA, and caffeic acid were effective at 100 *μ*g/mL; however, at 300 *μ*g/mL, the cell survival rate suddenly dropped to nearly zero, suggesting cell toxicity at this concentration, as shown in Figures 2(f) and 2(g). Therefore, we systematically examined the cell toxicities of the compounds, as shown in Figure 3. Chlorogenic acid, 3,4,5-triCQA, and caffeic acid exhibited cell toxicities at 300 *μ*g/mL whereas other compounds did not exhibit any serious toxicity.

### 3.4. Mechanism of The Antiviral Effect

**Figure 4:**
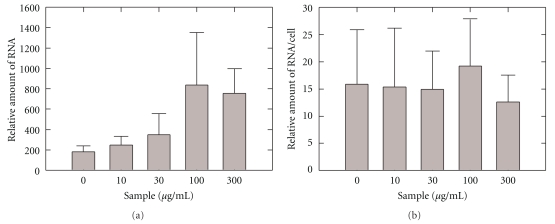
Real-time PCR assay of the PWE-induced antiviral state. (a): The relative amount of viral RNA in a culture supernatant. (b): The value of A per probability of survival in a WST-8 assay. Results are presented as mean value ± standard deviation; *n* = 6.

We measured the relative amount of viral RNA using real-time PCR.  Figure 4(a) depicts the amount of viral RNA as a function of the concentration of the PWE. The relative amount of viral RNA increased as a function of increasing PWE concentration, possibly due to an increase in the number of surviving cells. We then obtained the relative amounts of viral RNA per cell using a ratio of the relative amount of viral RNA and the cell survival rate, as plotted in Figure 4(b). Intriguingly, the relative amount of viral RNA per cell remained almost constant as the PWE concentration was increased (Figure 4(b)).

## 4. Discussion

Propolis is known to have several biological activities, including antimicrobial [30], antibacterial [31, 32], antiviral [7–20], and immunomodulatory effects [33–35]. In particular, the antibacterial and anti-viral effects of propolis have been known for a long time [36–38], and propolis has also been reported as an anti-influenza compound [21–24, 39]. 

Esanu et al. [22] reported that the slight anti-influenza effect of rutin was attributed to glucoside-induced vasodilation, which would favor the penetration of the virus into the blood stream at the level of the nasal mucosa. In another report [40], rutin and quercetin, which are both components of propolis, were reported to increase the HA titers and mortality rates of PR8-infected mice whereas NaF decreased both parameters. In this same report, caffeine and adamantane derivatives were reported to have anti-influenza activities.

The composition of propolis varies depending on the plant sources that are accessible to the bees [27, 29]. Inconsistencies have emerged among several reports using propolis from different sources, and, therein, these inconsistencies are most likely attributed to differences in the chemical properties of the propolis as a function origin. Here, we used the most popularly distributed type of propolis in Japan, Brazilian green propolis that is collected in the Minas Gerais state throughout this study. Brazilian green propolis-derived PWE primarily contains polyphenolic compounds, such as flavones, flavanones, phenolic acids, and phenolic acid esters, the compositions of which remain relatively constant (data not shown). It should be noted that the propolis used in this study does not include quercetin [41].

Here, we demonstrate that PWE and various caffeoylquinic acids that are contained in PWE can restore the viability of MDCK cells that have been infected with influenza virus in a dose-dependent manner. Although caffeoylquinic acid was reported to have an anti-influenza effect [42], our results provide evidence that 3,4-diCQA purified from propolis has a particularly potent anti-influenza activity. Our results also demonstrate that the cause of the anti-viral activity of the propolis can be attributed to caffeoylquinic acids. Additionally, we found that caffeic acid had an anti-influenza activity whereas quinic acid did not. Therefore, the caffeoyl group might be an indispensable moiety in the molecular structure of caffeoylquinic acids in terms of anti-influenza activity. Moreover, the other PWE component such as *p*-coumaric acid, artepillin C, baccharin, drupanin, and kemferide (not possessing the structure of caffeoylquinic acid) had not the anti-influenza activity,

Moreover, PWE and caffeoylquinic acid did not have any cytotoxic impact on the effective concentration range for anti-influenza activity.

It must be noted that the anti-influenza activity of PWE can be only partially explained by compounds tested here (ref. Tables 1 and 2). Thus it is highly possible that unknown efficient compounds must be included in PWE. However, the identification of such compounds is definitely a future task.

The primary interest of the paper is focused on the working mechanisms of the antiviral activities of these compounds. Therein, the initial question was whether the observed antiviral activities are exerted on the virus or the host cell. To address this, we measured the relative amount of viral RNA in the cultured cell with and without antiviral compounds. 

Because the relative amount of virus RNA per viable MDCK cell was not significantly different between groups with different compound concentrations, it is highly possible that PWE has no direct effect on the virus or does not interact with viral components. 

Although the quantity of mRNA for HA (hemagglutinin) relative to that of mRNA for glyceraldehyde-3-phosphate dehydrogenase in influenza virus-infected MDCK cells decreased to less than 10% in the presence of the anti-influenza agent ribavirin (5 *μ*g/mL), it never decreased in the presence of 100 *μ*g/mL of PWE (data not shown). 

These findings are consistent with previous reports that propolis extract had no direct impact on herpes simplex virus but may induce internal cellular changes that can affect the replication of the virus, for example, through the interaction with NF-*κ*B [43]. Li et al. [44] have reported that dicaffeoylquinic acids specifically bind to the gp120 [45] of RSV and inhibit the virus-cell fusion events in the early stage of the replication cycle and cell-cell fusion at the end of the replication cycle [46]. The neuroprotective and immunomodulatory effects of PWE are also well known [33–35]. Thus, the anti-influenza activity of PWE does not derive from an inhibition of virus replication, as is the case for a neuraminidase inhibitory drug, but, instead, may be due to another mechanism, such as an enhancement of cell resistivity via the activation or inactivation of unknown cellular processes. 

We do not claim the discovery of a novel anti-influenza drug candidate; however, further extensive study of the caffeoyl group may direct the development of future novel anti-influenza drugs. Therein, it is important to note that the natural products that are represented by propolis may have prophylactic or moderate anti-influenza virus activities. These observations are also important because the influenza virus can easily develop resistance to developed drugs, and it generally takes a long time for new drugs to be approved by the FDA. During the long development period, natural products may be able to satisfy treatment needs. Propolis may have general pharmacological value as a natural mixture and not as a source of new, powerful antiviral compounds [37].

Our study provides strong evidence that PWE has an anti-influenza effect. We have demonstrated that caffeoylquinic acids are the active anti-influenza components of PWE. By the combination analysis using the cell viability assay and real-time PCR, it is highly possible that PWE has no direct effect on the virus or does not interact with viral components. These findings could facilitate the application of natural products as prophylactics or moderate anti-influenza agents, as well as structural optimization based on caffeoylquinic acids and a further enhancement of our understanding of the intracellular process that occur during influenza virus infection.

## Figures and Tables

**Table 1:** Concentrations and molecular weights of the constituents of Brazilian green propolis [27, 29].

PWE components	Content (w/w%) in PWE	Molecular weight (g/mol)
Chlorogenic acid	2.7–3.6	354.3
Caffeic acid	0.2	180.2
3,5-Dicaffeoylquinic acid	4.3–4.9	516.5
3,4-Dicaffeoylquinic acid	3.3–6.1	516.5
4,5-Dicaffeoylquinic acid	—^#^	516.5
3,4,5-Tricaffeoylquinic acid	0.2	678.6
Quinic acid	—^#^	192.2

^#^: data not available.

**Table 2:** The EC_50_ of PWE components determined by curve fitting of the data in Figure 2.

PWE components	EC_50_ (*μ*M)
Chlorogenic acid	341.5^#^
Caffeic acid	191.2^#^
3,5-Dicaffeoylquinic acid	207.8^#^
3,4-Dicaffeoylquinic acid	81.1 ± 2.9
4,5-Dicaffeoylquinic acid	280.6^#^
3,4,5-Tricaffeoylquinic acid	114.6^#^
Quinic acid	>1561

^#^: standard error not deterministic.
